# Potential of Endophytic Fungi Isolated from Cotton Roots for Biological Control against Verticillium Wilt Disease

**DOI:** 10.1371/journal.pone.0170557

**Published:** 2017-01-20

**Authors:** Yuan Yuan, Hongjie Feng, Lingfei Wang, Zhifang Li, Yongqiang Shi, LiHong Zhao, Zili Feng, Heqin Zhu

**Affiliations:** State Key Laboratory of Cotton Biology, Institute of Cotton Research of Chinese Academy of Agricultural Sciences, Anyang, Henan, P. R. China; Tallinn University of Technology, ESTONIA

## Abstract

Verticillium wilt is a soil-borne disease, and severely limits the development of cotton production. To investigate the role of endophytic fungi on Verticillium wilt, CEF-818 (*Penicillium simplicissimum*), CEF-714 (*Leptosphaeria* sp.), CEF-642 (*Talaromyces flavus*.) and CEF-193 (*Acremonium* sp.) isolated from cotton roots were used to assess their effects against cotton wilt disease caused by a defoliating *V*. *dahliae* strain Vd080. In the greenhouse, all treatments significantly reduced disease incidence and disease index, with the control efficacy ranging from 26% (CEF-642) to 67% (CEF-818) at 25 days (d) after inoculation. In the disease nursery, compared to controls (with disease incidence of 33.8% and disease index of 31), CEF-818, CEF-193, CEF-714 and CEF-642 provided a protection effect of 69.5%, 69.2%, 54.6% and 45.7%, respectively. Especially, CEF-818 and CEF-714 still provided well protection against Verticillium wilt with 46.9% and 56.6% or 14.3% and 33.7% at the first peak of the disease in heavily infected field, respectively (in early July). These results indicated that these endophytes not only delayed but also reduced wilt symptoms on cotton. In the harvest, the available cotton bolls of plant treated with CEF-818 and CEF-714 increased to 13.1, and 12.2, respectively. And the seed cotton yield significantly increased after seed bacterization with CEF-818 (3442.04 kg/ha) compared to untreated control (3207.51 kg/ha) by 7.3%. Furtherly, CEF-818 and CET-714 treatment increased transcript levels for PAL, PPO, POD, which leads to the increase of cotton defense reactions. Our results indicate that seed treatment of cotton plants with CEF-818 and CET-714 can help in the biocontrol of *V*. *dahliae* and improve seed cotton yield in cotton fields. This study provided a better understanding of cotton-endophyte interactions which will aid in developing effective biocontrol agents for Verticillium wilt of cotton in futhre.

## Introduction

Verticillium wilt, caused by a soil-inhabiting fungus *V*. *dahliae* Kleb., is the most overwhelming or distressing disease of cotton, which can cause severe yield and quality losses [1]. Moreover, this disease has progressively increased in many regions and has become a serious obstacle to cotton production in China [2]. However, lack of upland cotton germplasm immune or highly resistant to the defoliating pathotype, only little progress has been made towards introduction of disease-resistant breeding [3–4]. In addition, there are no effective fungicides currently available for controlling the disease [5]. Use of biological control agents to control Verticillium wilt on cotton is a promising eco-friendly strategy [6]. Over the last two decades, rhizosphere bacteria, such as *Pseudomonas* spp. and *Serratia plymutica*, have been shown to be effective antagonists of Verticillium wilt [7–8]. Recent studies have indicated that endophytes colonize the internal tissues of plants and can improve plant growth and plant health [9–11].

Endophytes are defined as microorganisms that can be isolated from surface-disinfected plant tissues or extracted from within plants and that do not harm the host plants [12]. Endophytes may play many important beneficial roles in the metabolism and physiology of the host plant, including fixing atmospheric nitrogen [13], solubilizing phosphates [14], synthesizing plant-growth hormones [15], degrading toxic compounds [16], inhibiting strong fungal activity [17] and antagonizing bacterial pathogens [18]. This definition has been expanded to include fungi, bacteria and actinomycetes that spend either all or a period of their life cycle colonizing the symplastic or apoplastic spaces of asymptomatic living plant tissues [19]. The internal plant tissues provide a protective environment for endophytes, which colonize an ecological niche similar to plant pathogens, especially the vascular wilt pathogens [20]. There have few systematic studies on cotton endophytic bacteria that are antagonistic to *V*. *dahliae* and *Fusarium oxysporum* [21]. Li et al. [22] reported that species of endophytic bacteria isolates were diverse antagonistic to both *V*. *dahliae* and *F*. *oxysporum* pathogens, and some had growth-promoting potential. An endophytic bacterial isolate has been demonstrated to have efficient biocontrol potential of *V*. *dahliae* in greenhouse and field trials [11]. Previously, a preliminary study has been done using eighty endophytic fungi selected from 27 genera to evaluate their inhibitory activity against a highly virulent *V*. *dahliae* isolate Vd080 *in vitro*. Subsequently, four isolates of them, CEF-818, CEF-714, CEF-642 and CEF-193 were characterized and identified as *Penicillium simplicissimum*, *Leptosphaeria* sp., *Talaromyces flavus*., and *Acremonium* sp. respectively. [23]. In present study, we further investigated inhibitory activities of the four iaolates and evaluated their potential as *V*. *dahliae* biocontrol agents.

## Materials and Methods

### Strains and plant materials

Four endophytic fungi (CEF-818, CEF-714, CEF-642, and CEF-193) used in previous experimental were isolated from cotton roots (planted in Anyang, Henan province, China, 36°10′N, 114°35′E), and were identified as *Penicillium simplicissimum*, *Leptosphaeria* sp, *Talaromyces flavus* and *Acremonium* sp via morphology and ITS (internal transcribed spacer region) phylogenetic analysis [23]. The strongly virulent defoliating *V*. *dahliae* strain Vd080 was used to infect cotton, which was separated from the diseased soil in Xinji, Hebei province, China (37°56′N, 115°15′E). Two upland cotton (*Gossypium hirsutum*) cultivars, Jimian11 (susceptible) and Lumianyan21 (tolerant), were used in the tests.

A lint sample (15 g) from each plot was used for fibre qualities (fiber length, strength, micronaire, length uniformity and elongation) analysis by a HVI 900 fibre testing system in Supervision Inspection & Testing Center of Cotton Quality, Ministry of Agriculture (Anyang, Henan province).

### Fungi cultured

The fungi included *V*. *dahliae* and endophytic fungi were cultured using two methods.

Liquid culture medium method of fungi: the fungi strain (*V*. *dahliae* or endophytic fungi) was cultured in liquid Czapek-Dox mediumat 25°C in a shaker incubator at 150 rpm. The concentration of spore suspension was adjusted to 1×10^7^ conidia/mL with deionized water.

Solid culture medium method of fungi: The maize-sand (V/V = 1:1) medium was disinfected by high pressure steam at 121°C for 20 min. Five macro liters of fungi (*V*. *dahliae* or endophytic fungi) culture solution was inoculated into sterile maize-sand medium at 25°C under static culture to generate a final spore counts of 1×10^8^ conidia per gram of solid medium.

#### Greenhouse

The endophytic fungi were inoculated using two methods. (i) Cotton seeds were surface-disinfected with 1% NaClO for 1 min followed by washing triple in sterile water. The seeds were soaked for 12 h with the filtrate (1×10^7^ conidia/mL) from endophytic fungi cultured in Czapek Dox liquid medium, then dried and sown in paper pots (6 cm in diameter and 10 cm in height, made of newspaper and without bottoms) filled with autoclaved substrate (vol/vol, vermiculite, sand = 6, 4) [23–24]. (ii) The solid culture medium of endophytic fungi (1×10^8^ conidia/g) were mixed with the sterile vermiculite and sand (V/V = 6:4) for 10% volume [25]. Cotton seedlings with one true leaf were inoculated with *V*. *dahliae* Vd080 conidia suspension (1×10^7^ conidia/mL) following the method described by Naraghi et al. [25]. Three replicates were performed for each treatment with more than 50 plants.

#### Disease nursery

The disease nursery is a nature field that had not been covered with cotton in the last few years. And the solid culture medium of *V*. *dahliae* Vd080 was scattered on the field for 20 g/m^2^, then the soil was peaceful plowed.

The solid culture medium of endophytic fungi was scattered into seed furrows at 20g, 30g and 40 g per meter of furrow, and the sterile maize-sand medium was used as control. Then, the surface-sterilized cotton seeds were sown in the disease nursery to assay biocontrol efficacy of endophytic fungi. Disease nursery tests were performed by arranging randomized complete block design with three replicates, in which each plot had an area of 60 m^2^ with 8 m long rows, spacing of 0.75 m between the rows and a plant spacing of 0.25 m.

#### Heavily infected field

Heavily infected field (about 1 ha) was a contiguous cotton–growing region for over 10 years in Anyang (36°10′N, 114°35′E), Henan province, China, in which cotton have been suffering from serious Verticillium wilt disease.

Surface-sterilized cotton seeds were soaked in the filtrate from endophytic fungi cultured in Czapek Dox liquid medium, with the sterile Czapek Dox liquid medium as the control. Then the seeds were sown into the heavily infected field. Three replicates were performed per treatment with 8 m long rows, 075 m row spacing and 0.25 m plant spacing. The biocontrol efficacy of endophytic fungi were assessed from late June (squaring stage) to late July or early August (flower and boll stage) in 2015 and 2016.

### Biocontrol efficacy of endophytic fungi

The biocontrol efficacies of four endophytic fungi against *V*. *dahliae* were tested in a greenhouse, a disease nursery and a heavily infected field. Experiment in each condition was performed with three bio-replicates and repeats for each treatment were in triple.

A scale of 0–4 was used to classify plants according to the percentage of plant tissue affected by chlorosis, leaf necrosis or defoliation (0 = healthy plant or plant without symptoms, 1 = plant affected by 1–33%, 2 = 34–66%, 3 = 67–99%, 4 = dead plant). The disease incidence, disease index and percentage of protection were calculated according to [24] and described as follows,

$$\begin{array}{l} Disease\;incidence\;\left(\%\right)=\left[\left(n_{1}+n_{2}+n_{3}+n_{4}\right)/n\right]\times 100;\\ Disease\;index=\left[\left(0n_{0}+1n_{1}+2n_{2}+3n_{3}+4n_{4}\right)/4n\right]\times 100;\\ Protection\;\left(\%\right)=\left[Disease\;index_{\left(control\right)}-Disease\;index_{\left(treatment\right)}\right]/Disease\;index_{\left(control\right)}\times 100; \end{array}$$

Where *n*_0_–*n*_4_ were the numbers of plants with each of the corresponding disease ratings, and *n* was the total number of plants assessed.

### RNA extraction and quantitative real-time PCR

Total RNA from roots of 0, 12, 24, 36, 48, 60, 72, 84, 96 hpi (hours past infection) was extracted from roots using a modified cetyltrimethylammonium bromide (CTAB) method [26] and was stored at –80°C. A 1 μg aliquot of total RNA was used for first-strand cDNA synthesis with M-MLV reverse transcriptase (TaKaRa, Bio, Japan/Clontech Laboratories, Göteborg, Sweden) following the manufacturer’s instructions.

Quantitative real-time PCR (qRT-PCR) was performed to determine expression of three defense-related genes (Phenylalanine ammonia-lyase, PAL; Polyphenol oxidase, PPO; Peroxidase, POD). The gene-specific primers used for qRT-PCR are listed in Table 1, and the cotton UBQ7 gene was used as an internal control. The expression assay was repeated three times and each assay was performed with three independent technical repeats. Relative expression ratios of target genes were calculated from the standard equation [27].

**Table 1:** List of primers used for qRT-PCR.

1	GenBank accession No.	Sequences of prime
UBQ7	DQ116441.1	F:5′- GAAGGCATTCCACCTGACCAAC-3′
		R:5′-CTTGACCTTCTTCTTCTTGTGCTTG-3′
PPO	JQ345705	F:5′- CCGCATAACCATCACAAG-3′
		R:5′-ACTCTCATCACCTTCAACA-3′
POD	L08199.1	F:5′—ATATCCTTGTTCTGTCTGCTA-3′
		R:5′-CTCCTTCTACCGTCTCTTC-3′
PAL	JN032297.1	F:5′-GCTTCTTAACAACAACATCAC-3′
		R:5′- TCTCCATTAGGTCCAACAG-3′

### Data analysis

Statistical analyses were performed using the SPSS 19.0 (SPSS, Inc., Chicago, IL, USA). The means were compared by the least significant difference (LSD) test, and significance levels were set at 1%.

## Results

### Biocontrol efficacy of endophytic fungi

The four endophytic fungi had no effect on normal growth of cotton (Table 2). The plant height, root length, fresh weight and root weight of cotton seedlings had not significantly difference between seed treated with endophytic fungi and untreated control. The results showed that endophytic fungi did not affect the growth of cotton.

**Table 2:** Biomass of cotton seedlings with seed treatment by endophytic fungus.

Code No.	Plant height	Root length	Fresh weight	Root weight
**CEF-714**	10.5±0.1a	11.4±2.4a	7.0±0.9a	2.3±0.5a
**CEF-818**	11.7±0.6a	14.1±1.8a	7.2±0.4a	2.1±0.2a
**CEF-193**	12.4±0.5a	13.4±2.6a	8.3±0.8a	2.3±0.3a
**CEF-642**	11.1±1.1a	11.1±0.2a	7.2±0.2a	2.1±0.1a
**CK (Czapek)**	11.9±0.2a	14.8±7.4a	7.4±0.4a	2.1±1a

#### Greenhouse experiments

In the greenhouse, cotton seedlings with one true leaf were inoculated with *V*. *dahliae* Vd080. The beginning of disease symptoms on endophyte-treated seedlings was 2–3 d later than that on control seedlings, and the disease incidence along with disease index were lower than those of controls (Table 3). Notably, the symptoms of defoliation or death had been improved. At 18 d after inoculation with Vd080, the controlling effects of the four endophytic isolates were all > 50% on cotton Verticillium wilt, but the protective effect of CEF-642 reduced to 29.5% at 25 d. However, the controlling efficacy of CEF-818, CEF-714 and CEF-193 was stable, with 51.0% and 41.5%, 59.4% and 47.6%, and 58.8% and 52.4% at 18 and 25 d, respectively. Similarly, the controlling efficacy of CEF-642 decreased with 50.6% to 26.0% at 18 to 25 d after inoculation via the matrix inoculation treatment, respectively (Table 3). The other three endophytic isolates had constant bio-control effects against Verticillium wilt on cotton in the greenhouse. Particularly, CEF-818 and CEF-714 exhibited higher control efficacy with 70.3% and 60.7% at 18 d after inoculation, and 57.2% and 58.2% at 25d after inoculation.

**Table 3:** Biocontrol efficacy of endophytic fungi against Verticillium wilt on cotton in greenhouse.

Code No.	Soaked seeds						matrix inoculation					
	18 days after inoculation			25 days after inoculation			18 days after inoculation			25 days after inoculation		
	Disease Incidence (%)	Disease index	Control efficacy (%)	Disease incidence	Disease Index	Control efficacy (%)	Disease Incidence (%)	Disease index	Control efficacy (%)	Disease incidence (%)	Disease Index	Control efficacy (%)
**CEF-818**	41.4±4.0b	27.7±5.6b	51.0±2.7a	32.9±6.5b	28.7±3.6bc	41.5±8.1bc	13.6±6.1c	11.5±0.8d	70.3±2.8 b	24.4±4.4c	19.4±2.3de	60.7±23.9b
**CEF-714**	32.4±4.6c	23.0±4.5b	59.4±3.2a	27.3±7.2c	25.8±5.6c	47.6±5.7ab	19.7±3.6c	16.5±2.1cd	57.2±6.4bc	27.6±4.0bc	20.3±3.4cde	58.2 ±12.1b
**CEF-193**	39.2±6.7b	23.3±3.7b	58.8±3.1a	28.0±5.8c	23.1±4.3c	52.4±5.0a	27.9±3.2b	22.7±2.4bc	41.8±5.5d	31.6±4.6c	26.3±3.9bcd	47.0±23.5bc
**CEF-642**	36.9±4.9bc	27.7±6.2b	51.1±5.5a	36.8±7.8b	34.2±5.2b	29.5±3.7c	30.1±2.8b	19.6 ±4.2c	50.6±5.7c	46.6±4.9b	36.4±4.6abcd	26.0±8.3c
**CK (Czapek)**	58.8±6.2a	56.6±5.3a	/	51.6±4.4a	48.8±6.1a	/	42.8±7.0a	39.3±4.1a	/	55.2±7.2a	49.2±3.9a	/

#### Disease nursery experiments

The 20, 30 and 40 g/m of matrix with endophyte isolates was sprinkled into the seed furrow to assay their biocontrol efficacy against Verticillium wilt (Table 4). The biocontrol efficacy was greatest via the 20 g/m matrix. Compared to the controls (disease incidence of 33.8% and disease index of 31.5), the disease incidence of plants with use of CEF-818, CEF-193, CEF-714 and CEF-642 was 11.5%, 14.2%, 18.1% and 21.6%, respectively; and corresponding disease index was 9.6, 11.9, 14.3 and 17.1. Thus, CEF-818, CEF-193, CEF-714 and CEF-642 provided a protection effect of 69.5%, 69.2%, 54.6% and 45.7%, respectively (Table 4). However, the protection effects were not increased with an increasing dosage of endophytic isolates. Although all of the four endophytic isolates exhibited significant biocontrol efficacy against Verticillium wilt on cotton, CEF-818 and CEF-193 provided the best protection in the disease nursery.

**Table 4:** Biocontrol efficacy of endophytic fungi against Verticillium wilt on cotton in disease nursery.

Treatment	20g/m			30g/m			40g/m		
	Disease incidence (%)	Disease index	Control efficacy (%)	Disease incidence (%)	Disease index	Control efficacy (%)	Disease incidence (%)	Disease index	Control efficacy (%)
**CEF-818**	11.5±1.3c	9.6±1.6c	69.5±3.5a	10.7±1.3c	8.9±1.1c	62.4±2.2a	11.3±0.7c	9.0±1.0d	66.0±3.6a
**CEF-193**	14.2±0.6c	11.9±0.9c	69.2±1.4a	13.8±1.4c	11.8±0.8bc	50.2±1.7b	16.7±0.4c	13.6±0.8c	48.7±1.5b
**CEF-714**	18.1±2.0bc	14.3±2.0bc	54.6±2.8b	18.9±1.4bc	14.5±0.7b	38.8±1.5c	20.4±1.1bc	16.3±1.5bc	38.5±2.1c
**CEF-642**	21.6±2.5b	17.1±1.3b	45.7±3.1c	23.0±1.2b	18.3±1.4ab	23.2±3.7d	20.7±1.3bc	16.7±1.4bc	37.0±1.6c
**CK**	33.8±1.9a	31.5±3.8a	/	28.1±2.1a	23.7±1.5a	/	30.3±1.7a	26.5±2.2a	/

#### Heavily infected field experiments

**Fig 1:**
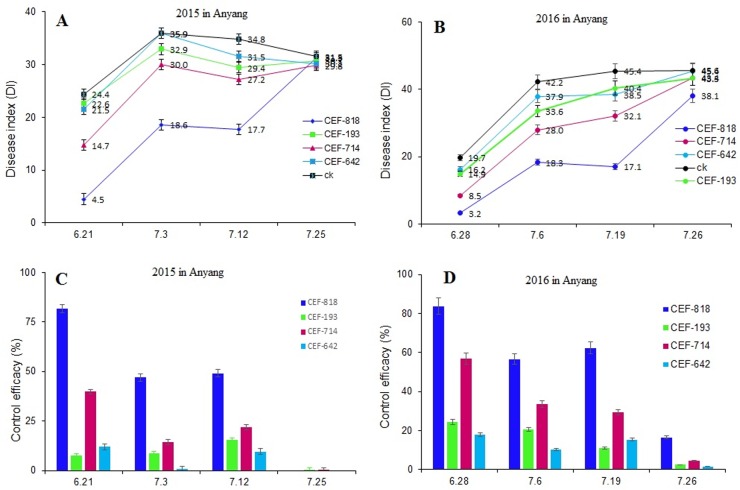
Endophytic fungi protects cotton plants from Vd infection in field. A: The disease index of Verticillium wilt on cotton; B: Biocontrol efficacy of endophytic fungi against Verticillium wilt on cotton.

We investigated the cotton wilt incidence during the squaring stage in heavily infected field (Fig 1). The disease occurrence and control of Verticillium wilt was similar in 2015 and 2016 (Fig 1). Compared to control, the disease indices were 4.5 and 3.2 with CEF-818 soaked-seed treatments at the early squaring period in 2015 (Jure 21) and 2016 (June 28) respectively, providing a protection effect of 81.6% and 83.8%. However, CEF-714 provided a protection effect of 39.8% and 56.9% in 2015 and 2016, respectively. In the period that followed, the protection effect was reduced; however, CEF-818 and CEF-714 still provided good protection against Verticillium wilt with 46.9% and 56.6% or 14.3% and 33.7% at the first peak of the disease (in early July), respectively. In addition, both fungi still had significantly decreased disease indices in mid-July. The CEF-818 could control against Verticillium wilt with 49.1 and 62.3% in 2015 and 2016, respectively. While CEF-714 could control against Verticillium wilt with 21.8% and 29.3% in 2015 and 2016, respectively. (Fig 1C and 1D), But CEF-193 and CEF-642 provided little protection (under 20%) against Verticillium wilt for cotton plants in the field. Unfortunately, the endophytic fungi did not provide any protective effect at the end of the squaring period, possibly because the colonization and population of endophytes was gradually diluted and weakened by competition with the ubiquitous microbial flora naturally present in soil [28].

In addition, four strains were selected for field and quality trials after evaluating their ability for disease suppression and growth promotion under heavily infected field conditions in 2016 (Table 5). The results showed that fiber quality parameters (fiber length, strength, micronaire, length uniformity and elongation) were not significantly affected by the seed bacterization with four endophytic strains under disease pressure (Table 5). Similarly, the boll weight and lint percentage were not significant difference between all treatments and untreated control. Compared to control, the available cotton bolls of plant treated with CEF-642, CEF-193, CEF-818, CEF-714 increased to 11.7, 12.0, 13.1, and 12.2, respectively. And there was a significance level by CEF-818 and CEF-714 treatments. The plants emerging from seeds bacterization (CEF-642, CEF-193, CEF-818, CEF-714) produced an average seed cotton yield of 3218.56 kg/ha, 3268.73 kg/ha, 3442.04 kg/ha and 3328.93 kg/ha. And the seed cotton yield significantly increased after seed bacterization with CEF-818 (3442.04 kg/ha) compared to untreated control (3207.51 kg/ha) by 7.3%. The increase of seed cotton yield by treatment with CEF-642, CEF-193, CEF-714 compared with the non-treated control ranged from 0.3%, 1.9%, 3.8%, respectively. While there was no statistical significance among any of the treatments.

**Table 5:** Effects of four endophytic fungi on Verticillium wilt on tolerant (Lumianyan21) cotton cultivars in a heavily infested field with *V*. *dahliae* at the Cotton Research Station during 2016 harvest season.

Treatment	Yield Traits				Quality Traits				
	Boll weight	Available cotton bolls	Lint percentage (%)	Yield (Kg•ha^-1^)	Fibre length (mm)	Uniformity (%)	Fibre strength (cN•tex^-1^)	Micronaire	Elongation rate (%)
**CEF-642**	6.2a	11.7bc	41.6a	3218.56b	29.2a	83.7a	28.8a	4.5a	6.7a
**CEF-193**	6.2a	12.0bc	42.5a	3268.73ab	28.8a	84.1a	29.4a	4.5a	6.8a
**CEF-818**	6.2a	13.1a	42.7a	3442.04a	29.9a	85.1a	29.2a	4.3a	6.9a
**CEF-714**	6.1a	12.2b	42.4a	3328.93ab	29.3a	85.0a	29.5a	4.5a	6.9a
**CK**	6.2a	11.5c	41.8a	3207.51b	28.7a	83.8a	29.1a	4.6a	6.7a

### Expression profiles of the related defensive genes

**Fig 2:**
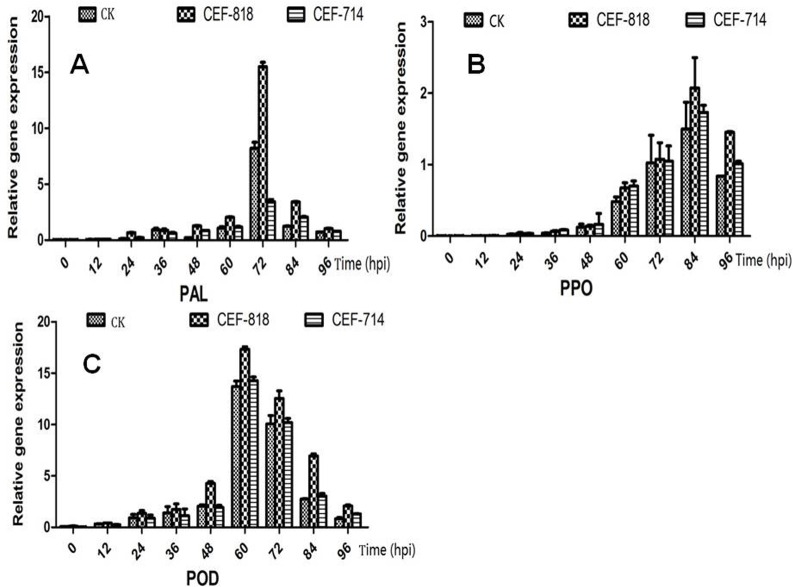
qRT-PCR analysis of three defense-related genes in cotton roots. A: *GhPAL*; B: *GhPPO*; C: *GhPOD*.

To further evaluate the role of CEF-818 and CEF-714 in cotton’s defense against *V*. *dahliae*, PAL, PPO and POD were analyzed by qRT-PCR in roots past infection by *V*. *dahlia* (Fig 2). Transcript levels for PAL were high in roots of CEF-818 treatment from 24 hpi compare to the control, with expression peak at the 72 hpi (Fig 2A). But the expression levels of PAL were erratic in CEF-714 treatment, which was showed considerably higher level at 48 and 84 hpi, but was lower at 36 and 72 hpi compare to the control ones, respectively (Fig 2A). It showed that the transcript levels for PPO were increasingly high in cotton roots of endophytic fungi treatment or control. But the expression levels of PPO were significantly higher in the roots of CEF-818 and CEF-714 treatment than that in the control roots, especially at 84 hpi by CEF-818 treatment (Fig 2B). While the expression levels of POD were not significant difference in roots by CET-714 treatment and control. And it was only slightly higher at 84 and 96 hpi in roots by CET-714 treatment than in the control. But transcript levels for POD were significantly higher in cotton roots by CEF-818 treatment than the control. Especially, it was more than two times at 84 hpi in the roots by CEF-818 treatment than the control (Fig 2C).

## Discussion

In a previous study, 642 endophytic fungi were obtained from 12 Verticillium-wilt-resistant cotton varieties, which were divided into 27 genera based on morphology and ITS (internal transcribed spacer region) phylogenetic analysis. Thirty-nine isolates exhibited potential fungi stasis against the pathogen at varying degrees. Among them, CEF-818, CEF-714, CEF-642 and CEF-193 were and identified as *Penicillium simplicissimum*, *Leptosphaeria* sp., *Talaromyces flavus*. and *Acremonium* sp. respectively. [23]. *T*. *flavus* was reported as an antagonist for control of Verticillium wilt in cotton, eggplant, potato and tomato [29–32]. Isolates in *Acremonium* spp. have been used as biocontrol fungi in management of root knot nematode disease [33–38]. There are some reports on *P*. *simplicissimum* and *Leptosphaeria* sp. as bioagents. For example, *Arabidopsis thaliana* grown in soil amended with barley grain inocula of *P*. *simplicissimum* GP17-2 or receiving root treatment with its culture filtrate exhibited clear resistance to *Pseudomonas syringae* pv [39]. In this study, we further explored their potential for biological control against cotton Verticillium wilt disease.

The biocontrol profile of endophytic fungi inhabiting plant tissues was not completely shown *in vitro* on PDA medium, but this was an effective method to screen and evaluate the potential of endophytes for biocontrol against Verticillium wilt in cotton. We assessed the antifungal activity of four endophytic fungi against *V*. *dahliae* Vd080 using dual culture and volatile and nonvolatile metabolite bioassays *in vitro* [23]. Further, the effectiveness of biocontrol against Verticillium wilt was validated in the greenhouse and the field by soaking seed and inoculation. Soaking seed was effective as a biocontrol method, which not only protects newly emerged seedlings but also enhances the ability of plants to resist biological and non-biological stresses [40]. Zhu et al. [24] reported that cotton co-inoculated with *V*. *dahliae* isolates CVn-WHg or CVd-WHw and CVd-AYb (a highly virulent isolate) had significantly less symptoms of Verticillium wilt compared with those inoculated with CVd-AYb alone in a greenhouse, suggesting that CVn-WHg and CVd-WHw protected cotton against *V*. *dahliae*. In the present study, the beginning of disease symptoms on cotton seedlings whose seeds had been soaked by endophytic isolates was 2–3 d later than for control seedlings. Meanwhile, the four endophytic fungi exhibited the potential to control Verticillium wilt by soaking seed and inoculation in the greenhouse. Particularly, CEF-818, CEF-193 and CEF-714 exhibited high and stable control efficacy at both 18 and 25 d after inoculation. Similarly, compared to controls (disease incidence of 33.8% and disease index of 31%), CEF-818, CEF-193, CEF-714 and CEF-642 provided a protection effect of 69.5%, 69.2%, 54.6% and 45.7% in the disease nursery, respectively. CEF-818 and CEF-714 also provided a protection effect of 81.6% and 39.8%, respectively, at the early squaring period in a heavily infected field.These results showed that CEF-818 and CEF-714 maybe has a potential for biological control against cotton Verticillium wilt disease.

In the harvest, fiber quality parameters (fiber length, strength, micronaire, length uniformity and elongation) boll weight and lint percentage were not significantly affected by the seed bacterization with four endophytic strains under disease pressure. But the available cotton bolls of plant treated with CEF-818 and CEF-714 increased to 13.1, and 12.2, respectively. And the seed cotton yield significantly increased after seed bacterization with CEF-818 (3442.04 kg/ha) compared to untreated control (3207.51 kg/ha) by 7.3%. The evidence showed that early disease control improved the survival rate of cotton bolls. It was result to the increase of seed cotton yield.

Furtherly, the expression of three defensive genes PAL; PPO; POD were analyzed by qRT-PCR in cotton roots by CEF-818 and CEF-714 treatment than the control after 48 hpi. The results showed that transcript levels for PAL; PPO; POD were significantly higher in cotton roots by CEF-818 treatment than the control. In plants, ROS (Reactive oxygen species) play a dual role as both toxic byproduct of normal cell metabolism and regulatory molecules in stress perception and signal transduction [41]. POD was an oxidative burst-related protein, and participated in the regulation of redox homeostasis in plant. It revealed that the accumulation of reactive oxygen species was relatively more in cotton cell by CEF-818 treatment, which leads to the increase of plant defense reactions [42–43]. In addition, PAL is an important gene in the phenylalanine metabolic pathway. It managed many important traits through the synthesis of secondary metabolites, including lignins and flavonoids. In addition, it is known to participate in salicylic acid-dependent signaling of the defense response to microbial pathogens [44]. *V*. *dahliae* penetrates into the host plant through the roots and spreads systematically through the xylem [45], so that lignin plays a critical role as a plant defense response. The expression of lignin synthesis-related genes is enhanced and lignin contents are improved in both resistant and susceptible cotton cultivars, but it is a greater extent in the more resistant cultivars [46]. Likewise, the extent of that lignification had being greater in tolerant cultivars than in susceptible plants infected with *V*. *dahlia* [47]. Similarly, lignin might be important at later infection stages, whereas the soluble and cell wall-bound phenolics seem to be involved in the early defense against the fungus [48], which could oxidize phenolic compounds into quinones to inhibit pathogen, and improve the resistance of plants.

In the field, the disease incidence and disease index of cotton significantly decreased at the squaring period with combined application of the respective four strains. However, there was no biocontrol of Verticillium wilt at the end of bud period. Similarly, strains that can be easily applied and readily colonize hosts under nursery conditions may not provide favorable effects for seedlings planted in the field [49–50]. Natural ecosystems are likely to host a stable and diverse population of root-associated microbes, and some of them may be considered true endophytes. Thus the colonization of some inoculated fungi is difficult likely due to competition with the ubiquitous microbial flora naturally present in the soil, consequently only a limited number of fungi have been tested for application under field conditions. It is difficult to predict how inoculated fungi will compete with the ubiquitous microbial flora naturally present in soil. If the inoculants are quickly competitively excluded, the initial growth promotion of the biocontrol fungi may be short-lived [28]. Despite this, the four endophytic fungi exhibited potential positive effects on Verticillium wilt of cotton *in vitro* and *in vivo*. Particularly, CEF-818 (*P*. *simplicissimum*) and CEF-714 (*Leptosphaeria* sp.) delayed and reduced the pathogen symptoms at the early squaring period in the field. This study represents a step toward understanding the ecology of endophytes as a means to develop effective biocontrol agents against Verticillium wilt of cotton.

## Supporting Information

S1 Data Supporting Information data. (XLSX)
